# Influence of vanadium on serum lipid and lipoprotein profiles: a population-based study among vanadium exposed workers

**DOI:** 10.1186/1476-511X-13-39

**Published:** 2014-02-24

**Authors:** Yang Zhang, Qin Zhang, Chengyong Feng, Xiaohui Ren, Hong Li, Keping He, Faxuan Wang, Dinglun Zhou, Yajia Lan

**Affiliations:** 1 Department of Occupational Health, West China School of Public Health, Sichuan University, No. 16, Section 3, South Renmin Road, Chengdu, Sichuan 610041, China; 2 Institute of Occupational Health Prevention, Panzhihua Iron and Steel Vanadium and Titanium Co., Ltd, Panzhihua, Sichuan, China; 3 School of Public Health, Zunyi Medical University, Zunyi, Guizhou, China; 4 Department of Occupational Health, School of Public Health, Ningxia Medical University, Yinchuan, Ningxia, China

**Keywords:** Vanadium, Lipid, Lipoprotein, Atherogenic index, Occupational exposure

## Abstract

**Background:**

Some experimental animal studies reported that vanadium had beneficial effects on blood total cholesterol (TC) and triglyceride (TG). However, the relationship between vanadium exposure and lipid, lipoprotein profiles in human subjects remains uncertain. This study aimed to compare the serum lipid and lipoprotein profiles of occupational vanadium exposed and non-exposed workers, and to provide human evidence on serum lipid, lipoprotein profiles and atherogenic indexes changes in relation to vanadium exposure.

**Methods:**

This cross-sectional study recruited 533 vanadium exposed workers and 241 non-exposed workers from a Steel and Iron Group in Sichuan, China. Demographic characteristics and occupational information were collected through questionnaires. Serum lipid and lipoprotein levels were measured for all participants. The ratios of total cholesterol to high-density lipoprotein cholesterol (HDL-C), low-density lipoprotein cholesterol (LDL-C) to HDL-C and apoB to apoA-I were used as atherogenic indexes. A general linear model was applied to compare outcomes of the two groups while controlling possible confounders and multivariate logistic regression was performed to evaluate the relationship between low HDL-C level, abnormal atherogenic index and vanadium exposure.

**Results:**

Higher levels of HDL-C and apoA-I could be observed in the vanadium exposed group compared with the control group (*P* < 0.05). Furthermore, atherogenic indexes (TC/HDL-C, LDL-C/HDL-C, and apoB/apoA-I ratios) were found statistically lower in the vanadium exposed workers (*P* < 0.05). Changes in HDL-C, TC/HDL-C, and LDL-C/HDL-C were more pronounced in male workers than that in female workers. In male workers, after adjusting for potential confounding variables as age, habits of smoking and drinking, occupational vanadium exposure was still associated with lower HDL-C (OR 0.41; 95% CI, 0.27-0.62) and abnormal atherogenic index (OR 0.38; 95% CI, 0.20-0.70).

**Conclusion:**

Occupational vanadium exposure appears to be associated with increased HDL-C and apoA-I levels and decreased atherogenic indexes. Among male workers, a significantly negative association existed between low HDL-C level, abnormal atherogenic index and occupational vanadium exposure. This suggests vanadium has beneficial effects on blood levels of HDL-C and apoA-I.

## Background

Vanadium is an inorganic trace element widely distributed throughout the lithosphere and biosphere [1]. Though vanadium is present in drinking water and a variety of foods, whether it is an essential trace element for human beings has yet to be established with certainty [2-4]. Due to its wide application in ferrous metallurgy, atomic energy industry, aircraft construction, and chemical industry, the chance of vanadium exposure, both environmental and occupational, has been increasing [1,5]. Therefore, a thorough understanding of possible beneficial and adverse effects from excessive vanadium exposure is critical.

Over the last several decades, biological actions of various vanadium compounds have been studied [6-8]. Most of the previous studies focused on the biological actions of vanadium compounds on improving glucose homeostasis and preserving insulin reserves [9-15], only a relatively small number of studies have associated vanadium exposure with altered lipid levels. Zychlinski [16] et al. found that long-term intratracheal administration of vanadium in rats was associated with pronounced decrease of the blood total cholesterol. Also, it has been demonstrated that vanadium significantly reduced triglyceride levels in experimental rats [10,15]. Despite the evidence available regarding total cholesterol (TC) and triglyceride (TG) levels, it is unknown whether vanadium is also related to other parameters of lipid and lipoprotein profiles and atherogenic indexes (TC/HDL-C, LDL-C/HDL-C and apoB/apoA-I). Besides, there is no sufficient information about the relationship between vanadium exposure and lipid and lipoprotein profiles in human subjects in literature.

On the other hand, however, studies have confirmed that lipid and lipoprotein abnormalities play a major role in the pathogenesis and progression of atherosclerosis, cardiovascular disease and diabetes mellitus which are becoming growing health problems worldwide [17,18]. Due to the increasing exposure facing around the world and the uncertain effects on lipid and lipoprotein, an in-depth study on vanadium-related lipid and lipoprotein changes become necessary.

So, by comparing the serum lipid and lipoprotein profiles of occupational vanadium exposed and non-exposed workers we undertook this population-based study to assess if serum lipid, lipoprotein profiles and atherogenic indexes changes were in relation to vanadium exposure.

## Results

As shown in Table 1, the average levels of HDL-C and apoA-I were higher in the vanadium exposed group than that in the control group (*P* < 0.05), no significant differences were observed between the two groups in TC, TG, LDL-C, non-HDL-C and apoB. Furthermore, TC/HDL-C, LDL-C/HDL-C apoB/apoA-I and HDL-C/apoA-I ratios were statistically lower in the vanadium exposed workers (*P* < 0.05).

**Table 1:** Concentration of various lipid and lipoprotein in exposed and control group

**Variable**	**Exposed group (N = 533)**		**Control group (N = 241)**		***P *** value*
	**Mean ± SD**	**Range**	**Mean ± SD**	**Range**	
TC(mmol/l)	4.28 ± 0.78	2.39-6.72	4.23 ± 0.78	2.18-6.88	>0.05
TG(mmol/l)	1.44 ± 0.92	0.30-6.94	1.47 ± 1.00	0.33-7.12	>0.05
HDL-C(mmol/l)	1.36 ± 0.32	0.64-2.42	1.23 ± 0.25	0.68-2.04	<0.001
non-HDL-C(mmol/l)	2.92 ± 0.78	0.69-5.44	3.00 ± 0.79	0.95-5.42	>0.05
LDL-C(mmol/l)	2.32 ± 0.52	0.93-4.08	2.32 ± 0.55	1.08-4.05	>0.05
apoA-I(g/l)	1.55 ± 0.32	0.85-2.81	1.12 ± 0.18	0.73-1.60	<0.001
apoB(g/l)	0.74 ± 0.16	0.26-1.18	0.72 ± 0.15	0.33-1.15	>0.05
HDL-C/apoA-I	0.34 ± 0.05	0.19-0.60	0.43 ± 0.08	0.24-0.73	<0.001
non-HDL-C/apoB	1.50 ± 0.15	0.93-2.58	1.60 ± 0.20	1.01-2.38	<0.001
TC/HDL-C	3.31 ± 0.94	0.52-7.09	3.55 ± 0.93	1.65-7.11	<0.001
LDL-C/HDL-C	1.81 ± 0.57	0.55-3.83	1.95 ± 0.58	0.84-4.53	<0.001
apoB/apoA-I	0.50 ± 0.15	0.18-1.32	0.65 ± 0.17	0.29-1.34	<0.001

*Covariance analysis adjusted for gender and age.

Further stratifying the study groups by gender, it became clear that the changes in HDL-C, TC/HDL-C, and LDL-C/HDL-C were more significant in male workers than in female workers (Table 2). In both female and male workers, the ratio of apoB/apoA-I was lower in the exposed group than in the control group (*P* < 0.05). There were no difference in the levels of TC, TG and LDL-C between exposed group and control group in subgroups according to gender (*P* > 0.05).

**Table 2:** Concentration of various lipids in exposed and control group in subgroups according to gender (Mean ± SD)

**Variable**	**Female (N = 175)**			**Male (N = 599)**		
	**Exposed group**	**Control group**	***P *** value*	**Exposed group**	**Control group**	***P *** value*
	**(n = 117)**	**(n = 58)**		**(n = 416)**	**(n = 183)**	
TC(mmol/l)	4.14 ± 0.71	4.00 ± 0.79	>0.05	4.32 ± 0.79	4.30 ± 0.76	>0.05
TG(mmol/l)	1.10 ± 0.57	0.96 ± 0.48	>0.05	1.54 ± 0.97	1.63 ± 1.07	>0.05
HDL-C(mmol/l)	1.47 ± 0.30	1.39 ± 0.23	>0.05	1.33 ± 0.32	1.18 ± 0.24	<0.001
non-HDL-C(mmol/l)	2.67 ± 0.30	2.61 ± 0.73	>0.05	2.99 ± 0.79	3.12 ± 0.77	0.047
LDL-C(mmol/l)	2.23 ± 0.50	2.15 ± 0.54	>0.05	2.36 ± 0.53	2.37 ± 0.54	>0.05
apoA-I(g/l)	1.61 ± 0.31	1.15 ± 0.15	<0.001	1.53 ± 0.31	1.11 ± 0.19	<0.001
apoB(g/l)	0.69 ± 0.14	0.64 ± 0.15	>0.05	0.76 ± 0.16	0.74 ± 0.15	>0.05
HDL-C/apoA-I	0.35 ± 0.05	0.47 ± 0.08	<0.001	0.34 ± 0.05	0.42 ± 0.08	<0.001
non-HDL-C/apoB	1.48 ± 0.14	1.56 ± 0.18	<0.001	1.51 ± 0.15	1.61 ± 0.21	<0.001
TC/HDL-C	2.92 ± 0.69	2.93 ± 0.62	>0.05	3.42 ± 0.97	3.75 ± 0.92	<0.001
LDL-C/HDL-C	1.58 ± 0.46	1.58 ± 0.43	>0.05	1.87 ± 0.58	2.06 ± 0.58	<0.001
apoB/apoA-I	0.45 ± 0.14	0.56 ± 0.12	<0.001	0.52 ± 0.15	0.68 ± 0.17	<0.001

*Covariance analysis adjusted for age.

Considering the limited number of cases in female, we employed the method of multivariate logistic regression to analyze data concerning the relationship of vanadium exposure and lower HDL-C, abnormal atherogenic index in male workers. Table 3 illustrates the results after adjusting for potential confounding factors such as age, smoking and drinking, occupational vanadium exposure was still associated with lower HDL-C (OR 0.41; 95% CI, 0.27-0.62) and abnormal atherogenic index (OR 0.38; 95% CI, 0.20-0.70).

**Table 3:** Multivariate analysis for low HDL-C level, abnormal atherogenic index and vanadium exposure among male workers

**Variable**	**Low HDL-C Level**		**Abnormal Atherogenic Index**	
	**Odds Ratio (95% CI)**	***P *** **value**	**Odds Ratio (95% CI)**	***P *** **value**
age (y)	1.01(0.98-1.04)	0.656	1.04(0.99-1.08)	0.142
Exposure	0.41(0.27-0.62)	<0.001	0.38(0.20-0.70)	0.002
Smoking	2.14(1.42-3.24)	<0.001	1.75(0.95-3.23)	0.074
Drinking	0.92(0.58-1.46)	0.729	1.19(0.60-2.38)	0.620

## Discussion

In this population-based study, we found that occupational vanadium exposure appears to be associated with increased HDL-C and apoA-I levels and decreased atherogenic indexes (TC/HDL-C, LDL-C/HDL-C and apoB/apoA-I). Furthermore, among male workers, exposure to vanadium was found to be significantly negatively associated with low HDL-C level and abnormal atherogenic index and this association cannot be attributed to confounding factors such as age, or unhealthy lifestyle (alcohol and tobacco consumption). This suggests vanadium may have beneficial effects on blood levels of HDL-C and apoA-I, which in a long term may reduce the risk of atherosclerosis.

Previous studies have demonstrated that a low plasma HDL-C concentration is a well-established risk factor for atherosclerosis and subsequent coronary heart disease, and several studies have reported higher concentrations of HDL-C in women compared to men [19-23]. According to our study, higher levels of HDL-C could be observed in the vanadium exposed group compared with the control group. What’s more interesting is when we stratified the study groups by gender, we found that compared with the control group, the exposed group had a much higher level of HDL-C in male workers, while in females, the levels showed no difference. A negative association still existed between vanadium exposure and low HDL-C level, independent of age, smoking and drinking. It seems that vanadium has a pronounced effect on male workers. Despite the well-documented gender difference and other identified metabolic factors responsible for the difference in HDL-C and apoA-I concentrations [20,21], it remains to be elucidated whether it is because of susceptibility of the men. ApoA-I is the primary protein component of HDL and is a direct protect factor against atherosclerosis independent of HDL-C [24]. ApoA-I facilitates selective uptake of HDL cholesterol by the liver by activating lecithin-cholesterol acyltransferase [25] and acting as a specific ligand for SR-BI [26]. In our study, we found higher apoA-I level and smaller HDL-C/apoA-I in the vanadium exposure group. This suggests vanadium may mainly increase apoA-I by increasing the synthesis or by inhibiting its catabolism and accordingly increase the apoA-I-rich HDL-C level. But the mechanisms about how vanadium influences the HDL-C, apoA-I and HDL-C/apoA-I need more researches.

Furthermore, since lipid is a well-established risk factor for cardiovascular diseases and diabetes, the relationship between lipid, lipoprotein profiles and vanadium may provide another explanation for the effect of vanadium treatment on cardiovascular function in the hyperinsulinemic, insulin-resistant conditions. High values of atherogenic indexes (TC/HDL-C, LDL-C/HDL-C and apoB/apoA-I) have been reported to be risk factors for cardiovascular disease that led to insulin resistant [27-31]. According to our study, atherogenic indexes values were lower in vanadium exposure group than in control group, which may be one possible mechanism of its insulino-mimetic and anti-diabetic effects. Among male workers, a significantly negative association existed between occupational vanadium exposure and abnormal atherogenic index after adjusting for age, smoking and drinking. Whether it is because of longer services or the susceptibility of men remains to be elucidated.

The question as to how exactly vanadium causes lipid and lipoprotein alterations remains elusive. Vanadate is reported to decrease plasma cholesterol and inhibit cholesterol biosynthesis and this effect is ascribed to a possible inhibition of oxidase [3], which may be important in mediating lipid effects induced by vanadium. Biomedical importance of vanadium confirmed by numerous studies is mostly based on its interaction with proteins, including enzymatic systems and cellar constituents [32-34], which may influence the lipid and lipoprotein synthesis or interfere with catabolism. Also, the stimulation of lipoprotein lipase activity by vanadate and stimulation of 2,3-diphosphoglycerate hydrolysis by vanadium may play a role as well [35,36].

This study finds that occupational exposure to vanadium in a low-dose, long-term exposure condition is associated with lipid and lipoprotein profiles alterations. One limitation of this study is that we did not have personal exposure measurements, which disabled us from further exploration of the relationship between lipids, lipoprotein levels and vanadium. In addition to occupational exposure measurements, biomarkers such as vanadium concentration in whole blood, serum, plasma or urine samples would be more accurate and convincing in terms of dose-response effects. Although it could be adjusted using statistical methods, higher prevalence of smoking and drinking in the exposed group could also be confounding variables. Lack of information on other factors known to influence lipid and lipoprotein levels such as levels of exercise and body mass index (BMI) may have an impact on the strength of the association. Also, because of the cross-sectional and observational nature of our study, results of this study should not be taken as evidence of a causal relationship. Future studies, preferably prospective cohort designs, are needed to clarify whether or not vanadium exposure leads to lipid and lipoprotein profiles changes, whether this effect is pronounced in certain subgroups such as the male workers and whether the association is dose-dependent. However, this study provides the initial epidemiological exploration demonstrating the association between vanadium exposure and altered lipid and lipoprotein profiles. The data may be of value for environmental exposure assessment of vanadium function.

## Conclusions

In summary, our findings suggest that occupational exposure to vanadium appears to be associated with increased HDL-C and apoA-I levels and decreased atherogenic indexes values (TC/HDL-C, LDL-C/HDL-C and apoB/apoA-I). Among male workers, a significantly negative association existed between occupational vanadium exposure and low HDL-C level, abnormal atherogenic index after adjusting for age, smoking and drinking. This suggests vanadium may have beneficial effects on blood levels of HDL-C and apoA-I.

## Methods

### Study population

All the participants in this cross-sectional study were selected from an Iron and Steel Group in Sichuan, China. The exposed group comprised of 533 workers who had worked in the vanadium-containing steel production workshop for at least 1 year, whereas the workers of the control group (241) were recruited from a cold rolling workshop in the same corporation that produced the steel products without vanadium.

The two workshops were established at the same time around 1990. The cold rolling workshop is located about 3.1 miles away from the vanadium production workshop. Vanadium production workshop mainly manufactures vanadium pentoxide, vanadium trioxide and ferrovanadium. Other than vanadium, these two factories suffer from similar occupational hazard factors such as noise, thermal radiation, etc. Routine occupational safety monitoring data indicated that time-weighted average concentration of vanadium smoke was 0.216 mg/m^3^ in the exposed group, and 0.013 mg/m^3^ in the control group. Workers in both workshops were trained by the same vocational training school. Both groups had similar socioeconomic status (salary, education, etc.) and background environmental factors (diet, place of residence, etc.). For the vanadium-exposed group and controls, the mean ages were 37.5 ± 6.6 years (range: 21-59) old and 36.1 ± 5.5 years (range: 22-57) old, respectively. The prevalence of alcohol consumption (38.8% vs. 11.2%; *P* < 0.001) and smoking (45.2% vs. 23.2%; *P* < 0.001) was significantly higher among the exposed than among the controls.

All participants gave written informed consent at recruitment. The Ethics Review Board of the Medical Faculty of Sichuan University approved the study.

### Main outcome measurements

Demographic characteristics and occupational information were collected through questionnaires. Blood samples were collected from an antecubital vein into vacutainer tube containing EDTA following a 12 h overnight. The concentrations of total cholesterol (TC) and triglyceride (TG) in the serum were determined by an enzymatic colorimetric method [37]. The HDL-C level was measured in the supernatant after precipitation of apoB containing lipoproteins with heparin-manganese chloride [38]. The concentration of low-density lipoprotein cholesterol (LDL-C) was determined according to the Friedewald formula [39]. ApoB was determined by a 2-site immunoradiometric assay and apoA-I by a competitive radioimmunoassay, using commercial kits from Pharmacia (Uppsala, Sweden). The coefficients of variation for all lipid parameters were <5%.

### Definition of terms

The ratios of TC to HDL-C (TC/HDL-C), LDL-C to HDL-C (LDL-C/HDL-C) and apoB to apoA-I (apoB/apoA-I) were used as the atherogenic indexes. The parameter HDL-C < 1.04 mmol/l was considered abnormal. Abnormal atherogenic index was defined as TC/HDL-C > 5, and/or LDL-C/HDL-C > 3, and/or apoB/apoA-I > 0.9.

### Statistical analyses

The R software from the “The Comprehensive R Archive Network” was used to perform all statistical processing. The results are presented as mean ± SD. Differences between groups were evaluated using analysis of covariance for continuous variables. Multivariate logistic regression model was used to evaluate associations between low HDL-C levels, abnormal atherogenic index and vanadium exposure, with odd ratios and 95% confidence intervals estimated. The significant level was 0.05 (two-tailed).

## Competing interests

The authors declare that they have no competing interests.

## Authors’ contributions

YZ, YJL and DLZ designed the study; YZ conducted the study and drafted the manuscript. DLZ and YJL participated in statistical analysis and revised the manuscript for important intellectual content and final approval. CYF and KPH conducted the study and involved in revising the manuscript. XHR, QZ, HL and FXW participated in the design of the study and involved in revising the manuscript. All authors read and approved the final manuscript.
